# An Overview of Nanomaterial Applications in Pharmacology

**DOI:** 10.1155/2023/4838043

**Published:** 2023-06-21

**Authors:** Madhura Roy, Arpita Roy, Sarvesh Rustagi, Neha Pandey

**Affiliations:** ^1^Centre for Translational and Clinical Research, School of Chemical and Life Sciences, Jamia Hamdard, India; ^2^Department of Biotechnology, Sharda School of Engineering & Technology, Sharda University, Greater Noida, India; ^3^School of Applied and Life sciences, Uttaranchal University, Dehradun, Uttarakhand, India; ^4^Department of Biotechnology, Graphic Era Deemed to Be University, Dehradun, Uttarakhand, India

## Abstract

Nanotechnology has become one of the most extensive fields of research. Nanoparticles (NPs) form the base for nanotechnology. Recently, nanomaterials (NMs) are widely used due to flexible chemical, biological, and physical characteristics with improved efficacy in comparison to bulk counterparts. The significance of each class of NMs is enhanced by identifying their properties. Day by day, there is an emergence of various applications of NMs, but the toxic effects associated with them cannot be avoided. NMs demonstrate therapeutic abilities by enhancing the drug delivery system, diagnosis, and therapeutic effects of numerous agents, but determining the benefits of NMs over other clinical applications (disease-specific) or substances is an ongoing investigation. This review is aimed at defining NMs and NPs and their types, synthesis, and pharmaceutical, biomedical, and clinical applications.

## 1. Introduction

Nanotechnology is considered to be one of the emerging and fascinating areas of science, and it enables researchers to design, manipulate, and derive the applications of substances/materials at a nanoscale [1–4]. The field of biotechnology plays a critical part in the transformation of biological and biomedical knowledge into industrial applications. Moreover, the unique physicochemical and biological characteristics of NMs have led to their applications in numerous sectors of health sciences. [5–8]. Thus, the versatile applications of NMs draw substantial and improvised growth in medical engineering [1, 9]. Over the past two decades, researchers and scientists are extensively working towards the synthesis and uses of NMs and NPs. Some NPs such as silver, gold, copper, platinum, zinc, and their oxides are demonstrated to be effective bioactive substances. [10, 11]. They are known to be excellent materials that exhibit applications in antibiotic therapies due to their extensive antimicrobial characteristics and less toxic effects. They are also employed in cancer therapy, and lately, NMs have shown increasing applications as anticancer and antimicrobial agents. [12]. As NMs are known for their biosafety; therefore, they are used in site-specific delivery and improved bioavailability of antigens. NMs are popularly used as adjuvants or carriers of cancer vaccines [13]. They assist in the transportation of the adjuvants and antigens at their targeted site [14], inhibit their degradation [15], and enhance the time of retention of the tumor antigens in the tumor and lymphatic tissues [16], thus, leading to the enhanced safety and efficacy of cancer vaccines. Natural products are tested for their therapeutic properties to treat various diseases like diabetes, microbial, cancer, inflammatory, and cardiovascular diseases since they exhibit distinctive benefits like fewer side effects and toxicity, effective curative characteristics, and are economical. But, some serious challenges like toxicity and biocompatibility are associated with the medicinal applications of natural products, as a result of which they are not able to clear the phases of clinical trials [17–19]. Compounds that are relatively large in size demonstrate various challenges like inefficient solubility, target-specific delivery, bioavailability, absorption in the body, in vivo instability, and ineffectiveness of formulation and adverse drug reactions; thus, novel drug delivery systems are a good option for effective transport of drugs at the targeted site of action [20, 21]. Nowadays, environment-friendly NPs are synthesized through green chemistry, which can also be used for various therapeutic applications, such as Youcef et al.'s synthesized green nickel oxide NPs with a modified glassy carbon electrode for sensing nonenzymatic glucose [22]. Li et al. synthesized environmentally friendly silver NPs from corn silk extract, exhibiting cytotoxic and antibacterial actions [23]. Das et al. synthesized carbon dots from *Apium graveolens*, which were demonstrated as effective hydrogels against bactericidal activities, and controlled release of phytomedicines [24]. García et al. synthesized magnetic NPs based on iron oxide from wastes of oranges, which had shown antibacterial activities [25]. Lyu et al. prepared mannose-coated serum albumin NPs which targeted neutrophils for the therapy of arthritis [26]. Pariona et al. had shown antifungal activities of green copper NPs against the *Fusarium solani*, *Neofusicoccum* sp., and *Fusarium oxysporum* species [27].

Therefore, nanotechnology has a significant contribution to the advancement of modern medicine and the effective synthesis of drugs, which resulted in enhanced release and delivery of drugs at their targeted site. It has enabled us to overcome the challenges of physical and biological sciences through the application of NMs in nanomedicine and nanotechnology-based drug delivery systems in various scientific areas [28]. The aim of this review is to discuss the existing concepts of NMs and NPs, their types, synthesis, and their recent clinical, pharmacological, and diagnostic applications.

### 1.1. What Are Nanomaterials?

NMs represent a technoeconomic sector that extends to various areas of applications and is becoming an interesting field for research and development. The physicochemical properties of NMs, like thermal conductivity, melting point, electrical conductivity, light absorption, scattering, wettability, and catalytic activity, have enabled them to exhibit effective results as compared to the bulk/large materials and have gained popularity in the field of technology and related advancements. The size of the NMs is measured in nanometers (nm) which is the SI unit (Système International d'Unités) and 1 nm = 1 × 10 − 9 meters. Primarily, NMs are known as substances that have a size of 1-1000 nm in a minimum of one dimension; nonetheless, they are frequently defined as having a diameter of 1-100 nm. Nowadays, in the European Union and the United States of America, there are various sections of legislation regarding NMs, but a specific definition of NM that is internationally accepted does not prevail due to the different hypotheses/opinions of various organizations [29].

Numerous organizations and agencies have defined or referred NMs as follows:

- *European Union (EU) Commission.* “A manufactured or natural material that possesses unbound, aggregated or agglomerated particles where external dimensions are between 1–100 nm size range” [30]
- *The Environmental Protection Agency (EPA)*. “NMs can exhibit unique properties dissimilar to the equivalent chemical compound in a larger dimension” [31]
- *The International Organization for Standardization (ISO).* “Material with any external nanoscale dimension or having the internal nanoscale surface structure” [32]
- *The US Food and Drug Administration (USFDA).* “Materials that have at least one dimension in the range of approximately 1 to 100 nm and exhibit dimension dependent phenomena” [33]

Deriving a universally accepted definition of NMs is a crucial challenge as there are numerous types of definitions that are proposed or used by different authorities; thus, it becomes a major obstacle for applying regulatory efforts for similar NMs [34].

### 1.2. Types of Nanoparticles

**Figure 1:**
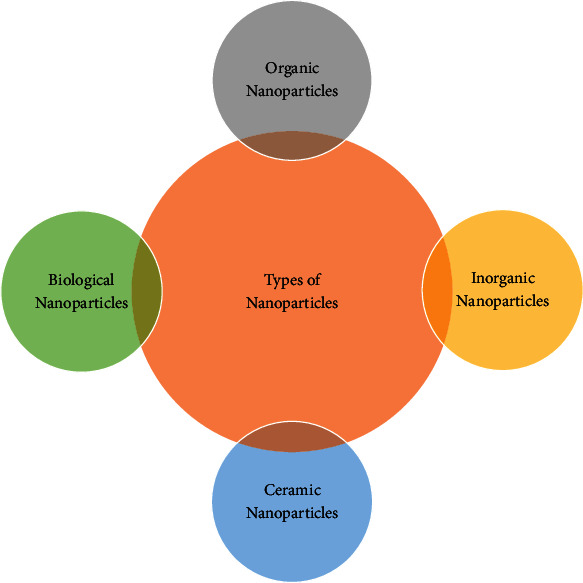
Types of nanoparticles used in pharmacological applications.

Some of the major types of NPs are discussed in Figure 1.

#### 1.2.1. Organic Nanoparticles

Liposomes, ferritin, dendrimers, and micelles are types of organic NPs. Organic NPs are hollow spheres (like liposomes and micelles) that are biodegradable and nontoxic [35]. Due to the aforementioned properties, they are the best options for drug delivery systems. The inner surface has matrix particles which are solid and form the overall mass, while the adsorption of other molecules takes place at the outer spherical region. The solid mass is encapsulated by the particles in the second case [36].

#### 1.2.2. Inorganic Nanoparticles

Inorganic NPs are nontoxic, hydrophilic, biocompatible, and devoid of carbon. They are more stable than organic NPs and can be differentiated into metal and metal oxide NPs.

#### 1.2.3. Metal Nanoparticles

Metals are utilized for preparing metallic NPs through constructive or destructive techniques. The precursors of metals are utilized for the preparation of metal NPs which demonstrates distinctive optoelectrical characteristics due to plasma on resonance characteristics. The size, shape, and facet determine the synthesis of metal NPs [37]. The NPs of all types of metals can be prepared [38]. The most common metal NPs are gold, silver, copper, aluminum, iron, zinc, lead, cobalt, and cadmium. NPs possess unique characteristics because of their surface characteristics (pore size, surface charge, ratio of the surface area-to-volume, and charge density of the surface), shapes (cylindrical, spherical, tetragonal, rod, hexagonal, and irregular), small size (10-100 nm), structure (amorphous and crystalline), and environmental and color aspects (air, sunlight, heat, and moisture) [39].

#### 1.2.4. Metal Oxide Nanoparticles

The metal oxide NPs are synthesized so that they can alter the characteristics of their corresponding metal NPs, like in the case of the oxidation of iron NPs to iron oxide NPs. The obtained iron oxide NPs are more reactive than iron NPs. Since the efficiency and reactivity of metal oxides are more enhanced as compared to their corresponding metals, thus, metal oxide NPs are preferred and synthesized [40]. Magnetite, zinc oxide, titanium oxide, iron oxide, silicon oxide, and cerium oxide are some of the metal oxide NPs.

#### 1.2.5. Ceramic Nanoparticles

They are also called nonmetallic solids which can be amorphous, dense, polycrystalline, porous, or hollow. Ceramic NPs can be synthesized by heating or simultaneous cooling [41]. Nowadays, these are the main areas of research because of their extensive applications in catalysis, imaging, photocatalysis, and photodegradation of dye [42].

#### 1.2.6. Bionanoparticles or Biological Nanoparticles

Biological NPs are a cluster of atoms or molecules which are synthesized in the biological environment and lie in the range of 1-100 nm and have a minimum of one dimension. Biological NPs are naturally present, and they are classified into 2 types, namely, intracellular and extracellular structures. Magnetosomes have an intracellular structure while liposomes have an extracellular structure. Some examples of biological NPs are lipoproteins, exosomes, viruses, magnetosomes, and ferritin [43].

### 1.3. Synthesis of Nanoparticles

**Figure 2:**
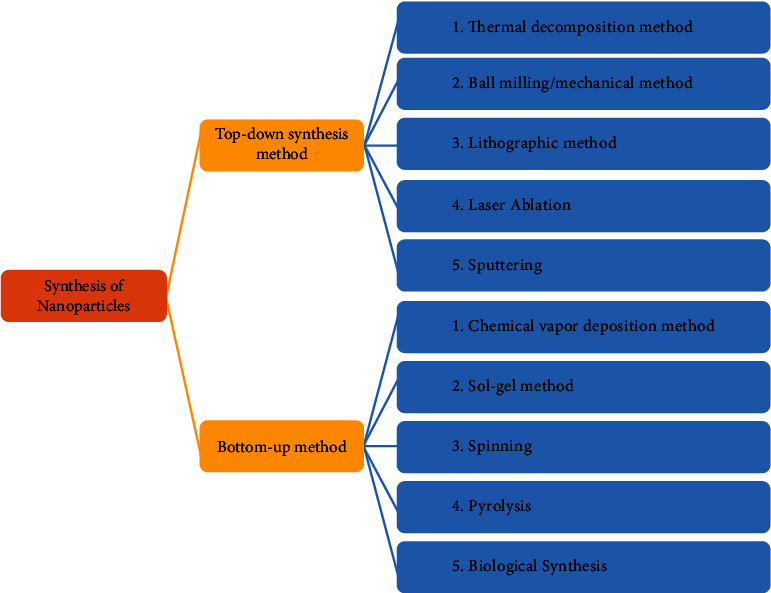
Different methods for synthesis of nanoparticles.

NPs can be manufactured in a number of ways, which can be divided into two categories: top-down synthesis and bottom-up synthesis (Figure 2, Table 1).

#### 1.3.1. Top-Down Synthesis Method

The destructive technique is utilized in the top-down synthesis method. The large substances are broken into small substances which are then converted to NPs. The destructive techniques employed in the top-down synthesis method involve milling, vapor deposition, and grinding [44]. The coconut shell NPs are prepared through this method. The synthesis of coconut shell NPs involved the milling technique, and the raw coconut shell was evenly ground at various time intervals through a ceramic ball and planetary mill. A study exhibited that the top-down method was utilized to synthesize magnetite, which has a spherical shape, from iron oxide [45]. The spherical particles of the colloidal carbon, whose size varies from 20 to 50 nm, are synthesized through this method. This method of synthesis was determined by the extent of polyoxometalates that are chemically adsorbed on the interfacial surface of the carbon. The carbon black gets clustered to form spherical-shaped smaller materials through adsorption. The micrograph exhibits that the particle size of carbon reduces, as the time of sonication increases. Through the integration of the sonication and grinding techniques, a sequence of transition metal dichalcogenide nanodots (TMD-NDs) was synthesized from larger substances [46].

*(1) Thermal Decomposition Method*. Thermal decomposition is an endothermal procedure where the heat is released by the decomposition of chemicals. The chemical bond of the substance is disintegrated through heat [38]. The decomposition temperature can be explained as the particular temperature that chemically decomposes the element. When the metals are disintegrated/decomposed at a particular temperature, they lead to the formation of NPs [47].

*(2) Ball-Milling/Mechanical Method*. It is the easiest, economical, and mechanical method of synthesizing NPs from large materials. The mechanical method synthesizes NPs through attrition. This method employs kinetic energy that is converted from the grinding medium to the particles, whose size is to be reduced. Particles with enhanced characteristics are synthesized through consolidation and compaction, which is an industrial procedure in which the NPs are “placed together”. By this method, alloys of various metals are synthesized.

*(3) Lithographic Methods*. This method enables synthesizing of most minute-sized micron particles, but it is an expensive and energy-intensive procedure. For many years, the technique of lithography is utilized for creating computers and printed circuits. Nanoimprint lithography is distinct from the conventional lithography method, and it resembles template synthesis. First of all, a template material is synthesized and then a pattern is formed by stamping a soft polymeric substance. Nanosphere lithography uses a latex sphere to synthesize templated matric. Lithography techniques are of several kinds like photolithography, soft lithography, nanoimprint lithography, electron beam lithography, dip pin lithography, and focused ion lithography. Photolithography is used for projection and proximity printing.

*(4) Laser Ablation*. It is a simple procedure employed for the production of NPs from various solvents. The irradiation through laser beams of various metals that are submerged in the solution condenses into a plasma that results in the synthesis of NPs [48]. This method is very beneficial and is distinct from the common chemical techniques for the synthesis of metals to NPs. The laser ablation method produces stable NPs that do not need any stabilizing substance.

*(5) Sputtering*. It is a phenomenon in which NPs are deposited through the ejection of particles from it [49]. The NPs are deposited as a minute layer through annealing, which is a very helpful technique. The size and shape of NPs can be detected through numerous factors like temperature, substrate, duration of annealing, and the thickness of the layer [50].

#### 1.3.2. Bottom-Up Method

This is a useful method where the NPs are synthesized from simpler substances, and this method is opposite to that of the top-down method. This method is classified into the following types: pyrolysis, spinning, biological synthesis, and sol-gel and chemical vapor deposition (CVD).

*(1) Chemical Vapor Deposition (CVD) Method*. In this process, on the surface of the substrate, a minute layer of gaseous reactant gets accumulated. The thin layer is deposited in a reactor vessel, where a chemical reaction occurs when the gas reacts with the hot substrate [51]. As a consequence of this reaction, a minute layer of the product is released on the substrate's surface, and this thin layer can be retrieved and then utilized. The benefits of the CVD method involve the synthesis of high pure, hard, uniform, and strong NPs while the drawbacks of this method involve the utilization of elaborate equipment and the production of toxic gaseous by-products [52]. Lee et al. [53] produced coated TiO2 through the chemical vapor deposition method, and the precursor used by them was titanium tetra isoperoxide (TTIP).

*(2) Sol-Gel Method*. It is a well-known and most utilized technique since it is the easiest bottom-up method for the production of NPs. In this technique, an appropriate chemical solution is used as a precursor, and conventionally, these precursors are chloride and metal oxide [54]. The precursor can be dispersed into the host liquid through various ways such as sonication, shaking, and stirring. The solution which is obtained has a solid and liquid phase which is isolated using numerous methods like sedimentation, centrifugation, and filtration to obtain the NPs. In this method, the conversion of a sol into gel includes hydrolysis and condensation reaction. The precursors that are utilized for the synthesis of TiO2 NPs are Ti(OBu)4 [55], Ti[OCH(CH3)2]4 (TTIP) [56], TiCl3 [57], and TiCl4 [58].

*(3) Spinning*. In this method, NPs are synthesized through a spinning disc reactor (SDR) that has a rotating disc through which physical factors like temperature can be controlled. Nitrogen or noble gases are infused inside the reactor to eliminate oxygen and avoid chemical reactions, and water along with the precursor is also poured into the reactor. The properties of NPs that are synthesized using SDR are characterized by numerous factors like the location of the feed, disc rotation speed, disc surface, liquid flow rate, and liquid/precursor ratio [59].

*(4) Pyrolysis*. It is a commonly used industrial technique that is employed for the synthesis of NPs. The precursor which is used in this method can be of liquid or vapor state and is burned in the flame, after which the precursor is shifted to a furnace with high pressure in order to obtain the NPs [60]. High temperature is preferred since it makes the process of evaporation easier, and the utilization of laser or plasma can produce high temperatures rather than the use of flames [61]. There are several benefits of the pyrolysis method; it is simple, efficient, and economical and is a continuous process with high production.

*(5) Biological Synthesis*. In this procedure, microorganisms (fungi and bacteria) and plant extract are utilized for the production of NPs. Phyto-nanotechnology uses an economical, easy, and ecofriendly synthesis of NPs, and mainly, they utilize plants for the synthesis of NPs. The primary benefit of phyto-nanotechnology is the production, scalability, and biocompatibility of NPs by using water as the reducing compound. The various components of the plant like leaves, seeds, roots, and stems are utilized for the production of NPs. The mechanism for the synthesis of NPs involving plants is yet to be defined. It has been exhibited that some types of NPs can be synthesized from proteins, vitamins, secondary metabolites, and organic acids like polysaccharides, flavonoids, terpenoids, heterocyclic compounds, and alkaloids. Microbes are also known as nanofactories as they involve ecofriendly, less energy, nontoxic, and economical techniques to synthesize NPs. Due to the presence of numerous reductase enzymes, microbes are used to detoxify and assemble heavy metals, and these enzymes cause the reduction of metal salts to NPs. For several years, microbes (fungi, bacteria, and yeast), metal-resistant genes, proteins, enzymes, reducing cofactors, and organic substances are utilized as reducing and capping substances for the production of NPs. The various benefits of using the biological or green method of synthesis are that it is safe, simple, ecofriendly, and cheap and has important uses in the health, pharmaceutical, and biomedical sectors.

*(6) Plant Extract*. Various parts of the plant like root, leaf, stem, and fruits are utilized for the production of metal or metal oxide NPs by utilizing the biological method, and it is also used to reduce and stabilize the NPs. By reducing metal salts, many biomolecules obtained from plants, including carbohydrates, proteins, and coenzymes, are employed to generate NPs.

*(7) Bacteria*. Various kinds of bacteria are used to generate novel and metallic NPs as they can reduce metal ions.

*(8) Fungi*. As fungi have intracellular enzymes which are utilized to generate metal/metal oxide NPs, the biological method for the production of NPs using fungi is a coherent method with well-defined morphology, and fungi produce more NPs as compared to bacteria [62].

### 1.4. Nanomaterial-Based Drug Delivery Platform

Nanomedicine is one of the disciplines of medicine that uses nanotechnology to avoid and treat numerous diseases by using nanoscale particles like nanorobots and biocompatible NPs [64], and it has multiple applications in sensory, diagnosis, delivery, and actuation functions in living beings [65–68]. Drugs that have less solubility exhibit numerous biopharmaceutical issues related to delivery like lack of bioaccessibility after ingestion via the mouth, lack of diffusion into the outer surface, large quantities needed for the administration through the intravenous route, and adverse effects after the process of conventional vaccination. But all these disadvantages are covered up by improvising the drug delivery system through nanotechnology. Synthesis of drugs at the nanoscale level is an extensive field of research and is considered the most advanced technique for the applications of NPs due to its significant benefits of altering certain characteristics like immunogenicity, drug release profiles, solubility, bioavailability, and diffusivity which can cause development and improvisation of biodistribution, routes of administration, fewer side effects, extended drug life cycle, and less toxicity [69]. Drug delivery systems with designed mechanisms are either targeted to a specific site or used to stimulate the distribution of therapeutic compounds to the site of action. Self-assembly, in which individual patterns or structures are produced using building blocks, is used in the synthesis of these medications [70]. Moreover, they have to combat some disadvantages such as sequestration or opsonization through the mononuclear phagocyte system [71]. The medications can be delivered by the nanostructures in two ways: passive and self-delivery. The medications are introduced into the inner cavity via the hydrophobic mechanism in the passive delivery system. Since less amount of the drug is encased in a hydrophobic structure, therefore, the expected quantity of the drug is released into the targeted site. [70]. In the self-delivery mechanism of delivery, the quantity of the drug that is intended to be released is directly combined with the carrier nanostructure for convenient delivery. In this method, the duration of drug release is very essential since the drug will not be delivered to the specific site and will quickly break down from the carrier, and its efficacy and bioavailability will be reduced if it is disintegrated from the nanocarrier [70]. The utilization of NMs or nanoformulations for the delivery of drugs can be differentiated into two methods: active and passive. In the active delivery system, substances like peptides and antibodies are combined with the drug delivery systems to attach them to the structures of the receptors expressed at the particular site. In a passive distribution system, the synthesized complex of the carrier of the drug is transported via the blood and is moved to the specified organ/site through various factors such as size, pH, temperature, and sites of the molecules. The primary targets are the proteins or antigens on the surface of the cell, receptors on the plasma membrane, and the components of the plasma membrane [72]. Nowadays, most of the drug distribution mechanisms that are driven by nanotechnology are targeting cancer disease and its treatment. Drug resistance is an ongoing challenge that poses threats to human beings and requires continuous development of antimicrobials. One such example is the opportunistic bacteria, *Pseudomonas aeruginosa*, which cause severe lung infections in immunocompromised individuals and exhibit drug resistance to conventional antibiotics. A specially designed drug delivery nanoassembly of lipid-coated mesoporous silica core-shell has been developed as an alternative to this challenge. It distributes antibiotics to both intracellular and extracellular surfaces of the bacteria, delays untimely drug release, and improves antimicrobial effectiveness. No discernible cytotoxicity was found. As a result, this lipid-coated targeted nanoassembly can be regarded as an effective antibiotic delivery system [73]. Makabenta et al. reported that by the process of innovative multimodal antibacterial pathways, NMs may delay or eliminate the emergence of drug resistance [74]. Currently, numerous massive research and developments in the area of drug distribution/delivery mechanisms have enabled the therapeutic compounds to reach their targeted site for the therapeutic intervention of various diseases [75, 76]. Some drug delivery systems are conveniently utilized, while some of them pose some disadvantages that are to be resolved, and more advanced methods/techniques are to be engineered for the better delivery/distribution of drugs to their specific site. Therefore, drug delivery systems that are designed using nanotechnology are being extensively researched for better therapeutic outcomes. Various biopolymeric NPs are used for drug delivery mechanisms. A few of them are listed below.

#### 1.4.1. Chitosan

It demonstrates mucoadhesive activities and is utilized in the tight junctions of the epithelial tissue. Thus, NMs derived from chitosan are extensively utilized for the prolonged production of drugs into many types of epithelia like pulmonary [77], intestinal [78], buccal [79], eye [80], and nasal [81]. Silva et al. [82] synthesized and examined the effectiveness of an isotonic solution (0.75% w/w) of HPMC (hydroxypropyl methylcellulose) having chitosan or hyaluronic acid or sodium tripolyphosphate NPs to deliver ceftazidime which is an antibiotic to the eye. The parameter of the rheological synergism is evaluated by enumerating the viscosity of NPs that are placed in proximity to the mucin in numerous proportions of mass. The least viscosity was noted when the NPs of chitosan were set in close contact with mucin. But the NPs exhibited mucoadhesion that caused effective interaction with the ocular mucosa and increased the release of antibiotics, and thus, the half-life of the drugs administered in the eyes is prolonged using the NPs. The NPs did not demonstrate any cytotoxic effects for 2 types of cell lines: HEK 239T and ARPE-19. Moreover, the NPs preserved the antibacterial functions, hence creating a significant formulation with enhanced mucoadhesive functions for the efficient use of ocular medications. Pistone et al. [83] synthesized NPs of chitosan, pectin, and alginate so that the drugs can be efficiently administered through the oral route. The drugs' biocompatibility was determined using the NPs' solubility in saliva, and their cytotoxic effect was determined using an oral cell line. The NPs of alginate are very stable for a minimum of 2 hours in the artificial salivary environment while that of pectin and chitosan were unsteady. But the chitosan NPs showed less toxicity, while the NPs of pectin and alginate demonstrated cytotoxicity in all the tested environments (time and concentration). The existence of zinc ions, which acts as an adhesive agent, might be the reason for the noticeable cytotoxic effects. Every drug possesses both benefits and drawbacks for release through the oral route; thus, it is necessary for further research and refinement.

#### 1.4.2. Alginate

It is a biopolymeric substance that has been extensively used for drug delivery. It has carboxyl groups and is differentiated as anionic mucoadhesive agents with effective mucoadhesive strength as compared to neutral and cationic polymers [84, 85]. Patil and Devarajan [86] synthesized alginate NPs consisting of insulin and nicotinamide, which are used as transfusion compounds to reduce the levels of serum glucose and increase the levels of serum insulin in rats with diabetes. NPs that are administered through a sublingual route (5 IU/kg) along with nicotinamide exhibit enhanced bioavailability (more than 80%) and availability pharmacology (more than 100%). NPs are effective insulin carriers through the sublingual route and have been verified in streptozotocin-induced mouse models with diabetes by acquiring increased bioavailability of 24.1% and high pharmacological potential of 20.2%, as compared to the injection (1 IU/kg) administered through subcutaneous route [86]. Haque et al. [87] synthesized alginate NPs in order to deliver venlafaxine (VLF) through the intranasal route to treat depression. The increased ratios of the blood-brain VLF concentrations to those of alginate NPs administered through the intranasal route compared to the VLF solution administered intravenously demonstrated the enhanced effectiveness of the NP formulation in direct delivery of the VLF to the brain. Thus, NPs in such scenarios are considered significant agents to treat depression.

#### 1.4.3. Xanthan Gum (XG)

It is a heteropolysaccharide that is of high molecular weight and is extracted from *Xanthomonas campestris*. XG exhibits effective bioadhesive functions and is a polyanionic polysaccharide. It is extensively utilized as a pharmaceutical agent due to its nontoxic and nonirritating characteristics [88]. Lafeur and Michalek [89] synthesized a carrier that was made of xanthan gum thiolated with l-cysteine to produce tannin for the treatment of sialorrhea in the buccal mucosa. Enhanced adhesion due to the thiolation of the xanthan gum on the buccal mucosa was observed in contrast to the traditional xanthan gum. Moreover, xanthan gum thiolate leads to increased uptake of saliva and tannic acid causes dryness of the oral mucosa, and thus, it will lead to reduced flow of saliva in patients suffering from sialorrhea. Huang et al. [90] synthesized hydrogels that were made of aldehyde-altered xanthan and carboxymethyl-modified chitosan having an angiogenic factor (antivascular endothelial growth factor, VEGF) and can be administered through injections to improvise the reconstruction of the abdominal wall. The hydrogel exhibits its activities in tissues like open wounds and digestive tracts. The VEGF that was present in the hydrogels was able to fasten up the process of angiogenesis and reconstruct the abdominal wall.

#### 1.4.4. Cellulose

The derivatives of cellulose cause changes in the gelation and solubility of the drugs which leads to its control release, and thus, it is extensively used in drug delivery systems [91]. Elsoud et al. [92] discovered the applications of nanocrystals of cellulose and chitosan NPs for the release of repaglinide (an antihyperglycemic PRG) via the oral route. The chitosan NPs exhibited an average distribution size of 197 nm, whereas the chitosan and cellulose nanocrystals formed hybrid NPs that possessed RPG. The chitosan hybrid NPs and cellulose nanocrystals that were oxidized had RPG with sizes ranging from 251 to 310 nm in diameter. The hydrogen bonds that were present between the drug and the cellulose nanocrystals caused the controlled release of the same, and consequently, the NPs that were synthesized using oxidized nanocrystals of cellulose exhibited reduced production in comparison to the NPs generated using the traditional nanocrystals of cellulose. Agarwal et al. [93] had synthesized a drug-targeting method on the basis of the amalgamation of alginate beads of calcium and carboxymethylcellulose (CMC) containing 5-fluoro acyl (5-FU) and is transported to the colon. The beads having reduced proportions of CMC caused enhanced much-adhesiveness and swelling in the simulated colonic region. Due to the presence of colonic enzymes, 5-FU was released (90%) from the enclosed beads. Hansen et al. [94] discovered 4 derivatives of cellulose, such as sodium carboxymethylcellulose, methylcellulose, cationic hydroxyethyl cellulose, and hydroxypropyl methylcellulose, for releasing formulations in the nasal mucosa. The relation of these derivatives was evaluated with a secondary ingredient. The pharmaceutical agent that was utilized in this method was acyclovir. The potentials of the polymers that were used as inactive pharmaceutical ingredients for their use in ciliary beat infusion and its administration via the tissues of the nostril cavity were evaluated. When the derivatives of cellulose were combined with a copolymer of graft polymers, there was an enhancement of the viscosity that was stimulated thermally. Moreover, the permeation into the nasal cavity was increased when acyclovir was coupled with cationic hydroxyethylcellulose. The derivatives of cellulose do not exhibit any adverse reactions on the nasal mucosa as per the evaluation of CBF.

#### 1.4.5. Liposomes

Alec Bangham was the first one who discovered liposomes in 1960. They are the most extensively researched carriers for drug delivery and are widely utilized in the cosmetic and pharmaceutical sectors for the delivery of numerous substances. They are synthesized using an engrained synthesis method to improvise the mechanism of drug delivery. They have sizes varying from 50 to 450 nm and are in the shape of a sphere made up of steroids and phospholipids [95]. As the structures of membranes of liposomes are analogous to the plasma membranes and they stimulate the permeation of drugs into the cell membrane, therefore, they are considered better agents for drug delivery [95]. It has been verified that liposomes can be utilized with hydrophobic and hydrophilic drugs due to their biodegradable and biocompatible properties and they are known to be steady therapeutic agents and enhance their biodistribution. There are 4 classes of liposomes, such as the following.

#### 1.4.6. Conventional Type Liposomes

They are composed of a bilayer that can be synthesized with cholesterol, anionic, neutral, or cationic phospholipids, and it contains a core material that is aqueous in nature. In such a scenario, the aqueous core material and the lipid bilayer can be packed with both hydrophilic and hydrophobic substances.

#### 1.4.7. PEGylated Types

In order to acquire the steric equilibrium, polyethylene glycol (PEG) externally permeates the liposomes.

#### 1.4.8. Ligand-Targeted Type

Ligands such as peptides, polysaccharides, and antibodies are linked to the liposomal membrane or to the terminal of the already linked chains of PEG.

#### 1.4.9. Theranostic Liposome Type

It is a combination of the aforementioned kinds of liposomes and has an NP with an imaging, targeting, and curative agent [96].

There are two ways in which a drug is loaded in the liposomes: active method (the drug is enclosed after the synthesis of liposomes) and passive (the drug is enclosed during the synthesis of liposomes) [97]. Hydrophilic drugs like 5-fluoro-deoxyuridine and ampicillin are specifically enclosed in the aqueous liposomal core, and therefore, their enclosure is not dependent on any changes related to the ratio of drug/lipid. But the hydrophobic drugs like indomethacin and amphotericin B were linked to the liposomal acyl hydrocarbon chain, and therefore, they are engulfed based on the acyl chain properties [98]. There are several disadvantages associated with the utilization of liposomes for the delivery of drugs in the form of opsonization and reticuloendothelial system; however, there are various aspects like improved retention effect and permeability that can be employed to stimulate the efficacy of drug delivery by liposomes [95, 97]. Preclinical studies of nano-based drug delivery platform for various diseases have been mentioned in Table 2.

There are several disadvantages associated with the utilization of liposomes for the delivery of drugs in the form of opsonization and reticuloendothelial system; however, there are various aspects like improved retention effect and permeability that can be employed to stimulate the efficacy of drug delivery by liposomes [95, 97].

### 1.5. Applications

#### 1.5.1. Cancer Vaccines

The terminology “cancer vaccine” describes a vaccination that either prevents individuals at risk from acquiring cancer-causing viruses or from developing cancer also called a prophylactic cancer vaccine or that treats cancer which has already developed also known as a therapeutic cancer vaccine [107]. In recent decades, nanotechnology has become increasingly prevalent in cancer vaccine research [108]. NPs are commonly used as cancer vaccine carriers or adjuvants because of their biosafety potential for target-specific antigen delivery and improvement of antigen bioavailability [109]. NPs can transport specific antigens and adjuvants [110], prevent or delay their degradation [111], and extend the retention period of tumor antigens in tumor and lymphatic tissues [112], thus improves vaccine safety and efficacy. Cancer vaccines demonstrate effective immune responses that initially need antigen-presenting cells (APCs), specifically dendritic cells (DCs) to engulf and release tumor-associated antigens (TAAs) [113]. Intradermal, subcutaneous, and intramuscular administration make cancer vaccines exposed to APCs [114, 115]. Because APCs have difficulty absorbing soluble proteins, antigen-loaded NPs that are similar to microorganisms are quickly detected and consumed by APCs, improving vaccine immunogenicity [116]. Recently, there are some NMs that have exhibited effective adjuvant or carrier functions in animal studies [117]. NP-based cancer vaccines demonstrate significant benefits over conventional cancer vaccines, such as that they do not cause degradation of the vaccines, stimulate the cytotoxic T lymphocytes, with the help of ligands, target the DCs, increase the antitumor activities by delivery of adjuvants, and enable controlled distribution and release [118]. Song et al. [119] conducted a preclinical study that utilized the NP-based delivery of antigens by coating PLGA NPs with a phospholipid membrane, and it resulted in a decrease in antigen-specific T cells, hence decreasing the metastasis of melanoma cells. Despite the enormous development of cancer vaccines and nanomedicines using NMs, as per the expert's opinion, cancer nanomedicine is aimed at addressing the fundamental drawbacks of traditional cancer screening and treatments. However, with the rise in interest and alluring qualities of nanotechnologies, there are still difficulties with their therapeutic use, leading some to assert that they are yet to achieve their full potential [120]. In this review, we discussed recent progress in nanotechnology for cancer vaccine delivery and design considerations for NPs as cancer vaccine carriers. Some of the types of cancer vaccines are discussed below.

#### 1.5.2. Tumor Cell Vaccines

Antigens can precisely activate T cells and B cells to generate a specific immunological response. Tumor-associated antigens (TAAs) and tumor-specific antigens are the two main categories of tumor antigens. TAAs are excessively expressed in tumor cells and persist in normal tissues but at a lesser level [121, 122]; thus, NPs are utilized to deliver these TAAs along with other adjuvants for immunotherapy [123]. Tumor cell vaccine is extracted from the tumor of an individual which is then lysed and treated in order to administer in another individual to evoke an immune response against a similar type of tumor (National Cancer Institute). The two types of tumor cell vaccinations are autologous tumor cell vaccines and allogeneic tumor entire cell vaccines. Autologous tumor cell vaccines are synthesized by obtaining a patient's tumor cells, converting them into vaccines in vitro, and then administering the resultant vaccine to the same patient [124]. Allogeneic tumor cell vaccines have two or three known human tumor cell variants and are designed to compensate for the drawbacks of autologous tumor cell vaccinations. [125]. The cancer cell membrane surface has various tumor antigens, and it is challenging to accomplish these activities using conventional synthesis methods, but it will result in the synthesis of an ideal vaccine [126]. As tumor vaccines have poor efficacy, they can only elicit a weak immune response; therefore, finding an effective addition to boost the vaccine efficacy of a tumor cell vaccination is critical [127]. To synthesize cancer cell membrane-coated NPs, Fang et al. [127] combined a pure type of tumor cell membrane with PLGA NPs (CCNPs). When monophosphoryl lipid A (MPLA) was coupled with CCNPs in a vaccine, it resulted in the development of dendritic cells to accept antigens and stimulate immune system activation. Likewise, Liu et al. [128] developed a multiadjuvant whole-cell tumor vaccine (WCTV) based on PLGA NPs modified with a cell-penetrating peptide, which resulted in GM-CSF and IL-2 distribution into tumor cells. Thus, the result obtained from these experiments demonstrated that the adjuvants of the substance led to the dendritic cell recruitment, release of antigens, and activation of T-cells.

#### 1.5.3. Dendritic Cell (DC) Vaccines

In tumor immunotherapy, the employment of DC in a DC cancer vaccine is identified as an effective procedure. A DC vaccine that is loaded with tumor antigens is recognized to possess effective functions for tumor immunotherapy [129]. But, in order to transport antigens to DCs, these vaccines primarily utilize viruses as carriers. Therefore, they release particular neutralizing antibodies and lead to an associated immune response, thus preventing the viral infection; hence, it has a risk-associated use [130]. Due to their distinct physicochemical functions, NMs have demonstrated effective abilities as vaccine adjuvants/carriers to increase the efficacy of antigens by enhancing the DCs to take up antigens, preventing the enzymatic degradation of antigens and controlling the immune cells [131]. Zeng et al. [132] designed a DC vaccine by inserting AFP1 cDNA in DCs using a signal peptide and AFP2 cDNA devoid of a signal peptide by incorporating calcium phosphate NPs and triggering progression with rmGM-CSF (recombinant mouse granulocyte-macrophage colony-stimulating factor). The in vitro and in vivo outcomes exhibited that the dendritic cell vaccine caused stimulation and expansion of T-lymphocytes to cause the production of the cytokine IFN-*γ*, which results in an enhanced immune response in the case of liver cancer. Moreover, Matsuo et al. [133] enclosed OVA in *γ*-PGA NPs (biodegradable NPs) and transported it in vitro to DCs. The outcomes of the in vivo experiment demonstrated that the utilization of *γ*-PGA NPs or OVA pulsed DCs leads to TAA-specific CTLs, causing an effective antitumor activity.

#### 1.5.4. Peptide Vaccines

Peptide vaccines that are used for the treatment of cancer exhibit benefits of high specificity to the immune response, lack of carcinogenic risk, direct stimulation, and no immunosuppression or autoimmune response and have more scope of applications [134]. But they also have some disadvantages like short half-life and low immunogenicity and might cause immune tolerance. In cancer peptide vaccines, NMs can act as carriers or vehicles for the cotransportation of immune antigens and adjuvants to stimulate the response mediated by the immune system, which ultimately enhances the immune activities of the peptide vaccine [135]. Zhuang et al. [136] employed LZnP NPs (lipid-coated zinc phosphate hybrid NPs) to transport the toll-like receptor 4 agonists and polypeptides (HGP10025-33 and TRP2180-188). Following the integration of H-2D (b) and H-2K- (b-) restricted particles, numerous epitopes became targets of specific MHC alleles, resulting in the immune system's recognition of the tumor. As a result, it was shown that the LZnP nanovaccines increased the number of CD8+ T cells and cytokine release, as well as IFN-. In comparison to a single peptide-loaded nanovaccine and free antigens, the nanovaccine's anticancer actions were effective in the prevention and treatment of melanoma-mouse models. [136].

#### 1.5.5. Genetic Vaccines

They are also recognized as nucleic acid vaccines, and they contain recombinant genes that encode a tumor antigen that is incorporated into vectors and then injected into the host's cells, where they produce exogenous antigenic proteins that stimulate an immune response against the antigen, preventing cancer. These vaccines are classified into DNA and RNA vaccines, with DNA vaccines receiving greater research attention than RNA vaccines. DNA vaccines are more effective for the treatment of tumors, but the traditionally used plasmid vaccines are less effective and exhibit safety concerns [137]. Synthetic nonviral novel vector substances are becoming an interesting area of research [138, 139]. Liu et al. [139] synthesized AC NPs (alginic acid-coated chitosan NPs) which can be delivered orally as a carrier of the legumain-DNA vaccine. The data obtained from the experiments demonstrated that the AC NPs are more effective than chitosan NPs (CNPs) in the prevention of the degradation of DNA in an acidic environment (pH = 1.5). Moreover, the outcomes also demonstrated that the vaccine can help in the protection of the DNA from gastric acid and caused significant uptake and expression of APCs, and effectively decreased the volume of tumors. Li et al. [139] utilized 4 strands of DNA that self-gathered into DNA nanostructures along with uniform sizes and distinct structures. The unmethylated CpG was base-paired to the ends of the DNA strands, yielding a 3-D DNA tetrahedra with an adjuvant. The studies exhibited that the nanostructures of DNA significantly penetrated without the transfection reagents into the macrophage-like RAW264.7 cells. The nanostructures of DNA have many advantages like precise structure, low toxicity, high plasticity, resistance to degradation by nuclease, and stable properties. Hence, they demonstrate an excellent opportunity to synthesize safe and effective carriers of nano vaccine.

#### 1.5.6. Nanomedicine to Treat Bacterial Infections

NPs demonstrate antibacterial actions or enhance the efficacy of antibiotics (nanobactericides). Metal NPs and their oxides and carbon-based NPs show antibacterial actions in various ways by suppressing the enzymatic activities, interfering with energy transaction and production of reactive oxygen species (ROS), and disrupting the synthesis of DNA [140, 141]. Consequently, numerous NPs such as silver NPs (AgNPs), gold NPs (AuNPs), Zinc oxides (ZnO), aluminum NPs, copper NPs (CuNPs), iron NPs, and chitosan NPs are extensively used to treat bacterial infections. NPs penetrate bacterial cell walls through passive diffusion [142, 143]. Antibacterial activities are passively induced by targeting the regulation of the morphology and physicochemical characteristics of NPs. It has been hypothesized that the production and target delivery of Ag ions have been shown to be stimulated through their morphological properties [144], which have been proven through the synthesis of AgNPs into various shapes such as spherical, disc, and triangular which were then tested against *S. aureus*, *Pseudomonas aeruginosa*, and *E. coli* by the disc diffusion procedure. Since the different shapes of NPs have different surface areas which determine the release of varied concentrations of Ag ions, consequently, the spherical AgNPs demonstrated the most potent activity against *E.coli*. Apart from the shape, size is also an important property for antibacterial actions [145]. Similarly, it has been demonstrated that NIR-assisted black phosphorus conjugated with ZnO and Au (Au-ZO-BP) causes the inhibition of *S. aureus* [146]. Despite the uniqueness of the passive modulation for targeting bacterial activities, it also possesses certain disadvantages like poor specificity and improper target delivery due to the stochastic mechanism of conjugation between the cells and the NPs, and it is dependent on the timings of circulation for drug delivery at the target cells [147, 148]. In order to overcome these disadvantages, an active strategy for targeting has been developed and extensively used for antibacterial treatment. It involves the employment of specific ligands to identify the corresponding substrates or moieties for better-targetted delivery of the antibacterial compounds to their targetted cells [149]. Due to their morphological and surface characteristics, the half-life of NMs can be increased in blood circulation causing enhancement of the drug concentration at the targetted tissues [150]. The cellular uptake of NMs can be stimulated by using ligands that have a high affinity for specific receptors or moieties, thus improving the therapeutic outcomes [151]. NMs can be bound to certain ligands like peptides, antibodies, small molecules, and aptamers to specifically identify the target cells [152]. Recently, Patel et al. developed a gold nanorod-based nanoassembly that destroys intracellular bacteria in combination with photothermal technology. This nanoassembly which is activated through a near-infrared responsive laser delivers the drug at its site of action along with photothermal therapy. This combination therapy enhances the efficacy of both drug delivery and antibacterial effects [153]. Another study by Bernardos et al. stated that the NMs based on mesoporous silica can demonstrate bactericidal actions by modifying their surface properties through grafting and cocondensation methods, providing numerous possibilities for developing and constructing antibacterial mechanisms, such as through the incorporation of widely recognized bactericides, by integrating the bactericides in the pores for distribution at the target site, or by including NPs known for their antimicrobial actions in the structure of the mesoporous silica [154].

#### 1.5.7. Nanoparticles in Diagnostics

Catalytic NPs are one of the widely studied applications for the detection of analytes. The use of LaCoO3 in ammonia detection was one of the premier applications [155]. These NPs have a major involvement in the process of catalyzation when ammonia was oxidized to NOx, which could be identified using the emission of light by luminol reaction. There are numerous NPs that have characteristics of peroxidase and can lead to the conversion of hydrogen peroxidase to radicals of hydroxyl. As a result, there are various investigations that reveal the detection of hydrogen peroxide by employing the NPs that act as peroxidase when linked to a substrate like 3,3,5,5-tetramethylbenzidine (TMB) or luminol [156, 157]. This procedure was chosen to determine various analytes that exhibited biological applications; for example, glucose peroxidase (one of the cofactors utilized by glucose) leads to the conversion of analyte into hydrogen peroxide, as detected by the aforementioned method [156]. Magnetic and metal NPs have been widely utilized for the detection of viruses [158, 159]. Due to their distinctive optical characteristics, AuNPs have been extensively employed in these viral detection approaches and are excellent candidates for quick colorimetric diagnostic testing [160, 161]. Over a time of 5 minutes, Ray et al. demonstrated the use of antispike antibody-attached Au NPs for the quick detection of a particular COVID-19 antigen or virus [162, 163]. Nanozymes are extensively used in immunoassay applications like in the capture-detection assays through antibody conjugation or targeting ligands and in enzyme-linked immunosorbent assays (ELISAs). Gao et al. [164] demonstrated that the NPs of iron oxide can be employed in an ELISA to determine the surface antigens of the hepatitis-B virus through a reaction of hydrogen peroxide and TMB, using a calorimetric method. Horseradish peroxidase was replaced by iron oxide NPs in ELISA. Simultaneously, the same group utilized the NPs of iron oxide that were enclosed in ferritin to determine the tumors (transferrin receptor positive) in the sections of tissue [165]. These NPs could specifically detect the transferrin receptor (present in excess amounts in the tumors) because of the presence of a coating of ferritin. The iron cores caused the catalyzation of 3,3′-diaminobenzidine (DAB) and hydrogen peroxide reaction to release the DAB-based polymers, which are of a brownish color. The researchers demonstrated that this method of staining can be employed for detecting various types of cancers in the tissue sections by staining the tumor cells with the brown-colored stain. Another method of fluorescent staining was used in which the ferritin was stained with fluorescein isothiocyanate (FITC) to identify the NPs that targeted the tumor cells. This procedure was able to differentiate normal cells from cancer cells due to high specificity and sensitivity. These procedures were employed in the detection of the Ebola virus [166], cancerous cells, single nucleotide polymorphisms, and various other targets. Duan et al. [166] synthesized a basic and cost-effective immunochromatographic strip assay on the basis of catalytic iron oxide NPs (MNPs) to detect the Ebola virus.

#### 1.5.8. Other Uses

*(1) Redox-Active Nanomaterials for Nanomedicine Applications*. Nanomedicine, which is one of the most widely studied fields of nanotechnology, utilizes the characteristics of NMs for biomedical applications like therapeutic delivery systems, diagnostic assays, and tissue engineering [167–170]. As NMs are extensively known and used because of their versatile characteristics like magnetic, optical, and thermal, the redox properties of NMs are also utilized due to their safe and efficacious biomedical applications [171]. One of the applications of NMs in the biomedical sector is their antioxidant functions especially in the scavenging of ROS (reactive oxygen species) [172]. ROS are redox-active compounds that are oxygenated and are released as byproducts of the metabolic activities in the body or are extracted from the surrounding [173–175]. Examples of reactive oxygen species are singlet oxygen (O2), hydrogen peroxide (H2O2), superoxide (O2•−), and highly reactive hydroxyl radical (•OH) [176]. Although ROS are extensively utilized as signaling agents throughout the body [177], they can also degrade biological compounds like DNA, lipids, and proteins [173]. An increased level of oxidative damage and ROS can cause oxidative stress which leads to adverse effects like cardiovascular disorders, cancer, and neurodegenerative disorders [178–180]. To overcome the severe reactions caused due to oxidative stress, various antioxidative methods are used which optimize the levels of ROS [181]. Examples of such antioxidants are glutathione peroxidase (GPx), superoxide dismutase (SOD), and catalase (CAT) families of enzymes [175, 176, 181]. Although GPx and CAT catalyze the breakdown of H2O2 into H2O and SOD distinctly catalyzes the dismutation of O2•− into H2O2 [175, 176]. In the scenario of glutathione peroxidase, glutathione also known as GSH, which is a selenium-carrying substance, is converted to glutathione disulfide (GSSG) through oxidation. The NMs which are antioxidants exhibit ROS scavenging through mechanisms similar to the antioxidative mechanisms of the human body and, therefore, are known for possessing enzyme-mimetic functions [182, 183]. NMs that demonstrate redox activities can be prooxidative too, causing the release of ROS. The surplus release of ROS can cause the destruction of the abovementioned mechanisms of the antioxidants, stimulating the enhancement of oxidative stress which can lead to the aforementioned severe reactions [184, 185]. But the prooxidative redox characteristics demonstrate various uses like in the treatment of cancer (which is linked to oxidative stress) by photodynamic therapy (PDT) or via releasing ROS. PDT employs an agent which is photosensitizing (PS) whose activation occurs through light, and then, it is transposed to the site of the tumor [186]. Particularly, the laser light of an appropriate wavelength is used for the excitation of localized PS to form a single photosensitizer (PS^∗^) with an excited state [187]. After which, the PS^∗^ goes through an intercrossing between systems to synthesize a triplet excited state (PS^∗∗^), which can (i) stimulate the transfer of electrons to the neighboring area and release ROS (especially O2•− or •OH, which are free radicals) indicated as type I or II or, (ii) go through the procedure of transfer of energy with the ground state (3O2) to form singlet oxygen (O2), indicated type II [187]. Thus, these ROS which are generated by NMs can then destruct the essential biomolecules of the tumor, which act as cytotoxic substances if especially applied to cancer cells. The widely studied NMs that are widely utilized in the sector of nanomedicine include gold, iron, silver, titanium metal oxide NPs, nanoscale allotropes of carbons (fullerenes, carbon nanotubes, graphene, and their derivatives), and cerium.

*(2) Nanomaterials Used for Biofilms*. Biofilms are an aggregation of bacteria that are strongly attached to a surface and are enclosed in a hard extracellular matrix composed of polymeric compounds; thus, it is difficult to eliminate them. Biofilms cause various types of infectious diseases in human beings [188]. Andre et al. [189] analyzed the antimicrobial properties of nanowires of vanadium pentoxide. They discovered that these nanowires resemble the functions of haloperoxidases in which they cause the catalyzation of hydrogen peroxide and bromide ions to form hypobromous acid. The nanowires used laboratory incubations to prevent the bacterial cells' growth, and they also inhibited the growth of biofilms on a ship in the Atlantic Ocean [190, 191]. Eventually, Gao et al. [192] examined the hydrogen peroxide and iron oxide NPs (which act as a peroxidase mimetic) combination as an antibiofilm substance. The increased rate of the degradation of hydrogen peroxide and the production of reactive substances by the NPs led to the destruction of the matrix constituents (killed implanted bacterial cells and polysaccharides) of the biofilm. In a follow-up experiment, the antibiofilm properties of the combination of hydrogen peroxide-iron oxide were observed in detail [193]. This experiment demonstrated that the NPs which are also known as CAT-NPs combine with the biofilms and exhibit active site catalyzation. The activities of the peroxidase were dependent on pH and exhibited enhanced catalyzation at an acidic pH of 4.5 and showed minimum effects at a neutral pH. Pathogenic biofilms have an acidic pH and CAT-NPs stimulate the release of free-radical from the hydrogen peroxide which consequently destructs the components of the matrix and leads to an absolute elimination of the bacterial cells in the biofilm. In vivo experiments of a biofilm of rodent model demonstrated that the regular administration via the topical route as utilized in clinical applications can significantly decrease the occurrence of tooth decay, leading to a novel therapy for oral diseases caused due to biofilm [193]. Moreover, the activities that depend on the pH cause the prevention of catalysis at the neutral pH and release of free-radical, causing in vivo biocompatibility [193]. The NPs of gold when combined with graphite carbon nitride (g-C3N4) exhibit effective bactericidal activities against *E. coli* and *Staphylococcus aureus* (which are drug-resistant), when integrated with hydrogen peroxide [194]. This combination of enzymes demonstrates in vitro antibiofilm activities and enhances the in vivo healing of wounds while eliminating the infections caused due to bacteria. The gold NPs, along with Ce (IV) centers that were restricted on the surfaces of SiO2/Fe3O4 shells or core NPs, exhibited DNAse-like enzymatic functions. These NPs caused the cleavage of DNA, which destroyed the in vitro-preformed biofilms and prevented the formation of biofilms [195].

*(3) Nanomaterials for the Prevention of Cancer*. Apart from the catalytic activities and antibiofilm therapies, the NPs of iron oxide demonstrate antitumor activities. In a study published in 2016, Zanganeh et al. found that incubating malignant cells with macrophage cells and iron oxide NPs (also known as ferumoxytol) resulted in increased cancer cell apoptosis when compared to incubation with just ferumoxytol or macrophages. This occurrence was because of the enhanced polarization of the macrophages to the proinflammatory phenotype, causing them to act against the cancerous cells. The alteration in polarization was caused by the enhancement of the reactive species of oxygen released by macrophages, whose synthesis was stimulated by the NPs of iron oxide. The researchers found that the growth of the tumor was decreased when the cancerous cells were injected prior to or post or at the same time as ferumoxytol. Antitumor therapy has an important issue in the distribution of therapeutic agents into cancer cells without harming healthy cells. A study demonstrated that the liposomal hybrid gold nanoassembly in addition to better photothermal activity was effective for the therapy of lung cancer. The main constituents of the nanoassembly comprised mesoporous silica shell-coated Au nanorods which provide an exceptional loading surface for doxorubicin encapsulation. IR 780 dye was used within the liposomes which were thermosensitive in order to improve the photothermal activity; then, the nanoassembly was encapsulated in those liposomes, after which folic acid and GE-11 peptide were combined on the liposomal surface, which led to the synthesis of the nanoassembly to be used for the synthesis. This dual-target strategy improves cellular actions by ameliorating the distribution of nanoassembly in cancerous cells which enhance the production of folate and epidermal growth factor. Irradiation using a near-infrared laser can stimulate the destruction of the nanoassembly, which enables the controlled delivery of doxorubicin at specific sites of action. Moreover, this dual-target therapy has been exhibited to be eleven times more potent than chemotherapy alone. This study shows that nanoassembly causes the apoptosis of cancerous cells by an intrinsic mitochondrial pathway which is stimulated by the chemo-photothermal therapy. This investigation presents a compelling prospect for a highly promising combination therapy for cancer [196].

**Figure 3:**
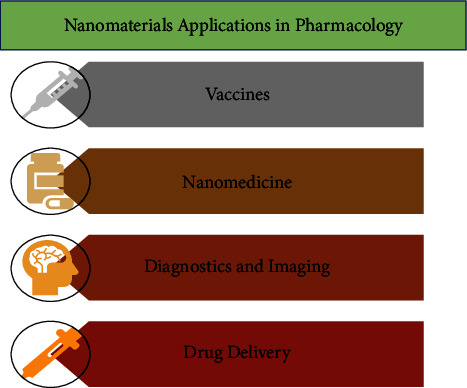
Nanomaterials applications in pharmacology

*(4) Nanomaterials for Disease-Specific Application*. In the case of diabetes, the *β*-cells of the pancreas exhibit impaired secretion of insulin [197]. The traditional therapeutic procedure involves the administration of either injectable or oral forms of insulin to overcome the lack of production of insulin or by suppressing hepatic gluconeogenesis. But the traditional marketed therapies/medicines that have been used for years have started exhibiting resistance to maintain the levels of glucose in the blood and cause various adverse reactions, like in the case of metformin, which is extensively marketed and demonstrates certain side effects like weight loss, diarrhea, and deficiency of vitamin B-12 in the body. Nonketotic hyperglycemia is one of the side effects of type-2 diabetes, but it can be resolved by employing metallic NMs because of their distinct physicochemical modified tribological characteristics to enhance the oxidative stress levels for the therapeutic management of diabetes [198]. Metallic NMs are known as scavenging substances as they mimic catalase (CAT), peroxidase, and oxidative functions which release free radicals and reduce inflammation and the ROS levels in the body. The metallic NMs are altered with various properties like peptides, ligands, antibodies, alloys, potent drugs, and DNA/RNA target cells to combine with the cell surface. The tribology of metallic NMs enables the combination of the surfaces of the metal-protein-cell which catalyzes the response of the enzymes to diseases. Metallic NMs also enhance the specificity, and bioavailability and cause the least side effects by decreasing the dosing regimen. Encapsulation of insulin in a matrix of zinc-silica for the safe and intended release of drugs is one of the procedures for the therapeutic treatment of diabetes. The presence of silica in the matrix does not cause aggregation and denaturation, and the zinc oxide leads to the consistency of the formulation. Furthermore, the presence of Zn ions during the synthesis and storage of insulin enhances insulin stability [199]. Trace metals like chromium help in the better management of type-2 diabetes. The tribology of chromium enables its site-specificity and overcomes various obstacles that stimulate the combination of chromium with the surface of *β*-cells because of the large ratio of surface area. Chromium can be used as a supplement and can enhance the pharmacological activities on the surface of *β*-cell [197]. Metals such as zinc and vanadium can be given like inorganic salts to maintain the glucose level in the plasma. Vanadium-ligand organic complexes exhibit enhanced lipophilicity and solubility and decreased toxicity. The NPs that are based on cerium-oxide demonstrate catalytic characteristics similar to that of catalase and SOD, because of this, they are extensively studied and widely used [200]. These catalytic properties exhibit antioxidative effects due to which they are used for the in vitro protection of the cardiac progenitor cells and neurons from oxidative stress [201, 202]. These clinical applications resulted in the utilization of cerium oxide for decreasing the chances of ischemia in stroke [203], treatment of retinal degeneration [204], and the coatings of the stent [205]. The catalase-like characteristics of these NPs were verified through in vitro testing. Several doses of these NPs were administered to the rats using injectables after the occurrence of stroke, and it was observed that the infarct size was drastically less when the rats were administered cerium oxide NPs or ceria, and the death of cells was also low [203]. In the infarct sites which were treated with cerium oxide NPs, the lipid peroxides were relatively low; thus, it can be inferred that the therapeutic result was achieved through oxidative stress. Likewise, the nanodots of molybdenum sulfide have demonstrated protective activities against ionizing radiation by using their antioxidative characteristics [206]. Bandages that have graphene quantum dots exhibit enhanced healing of wounds because of the peroxidase-like function of graphene that leads to antibacterial functions [207]. The NPs of iron oxide demonstrate antiaging and cardioprotective properties [208, 209]. Xiong et al. [208] discovered that the NPs of iron oxide provide protection from ischemic destruction in a model of reperfusion and ischemia. Its activity is not well-defined yet, but suggestively, it is caused because of the catalytic properties of NPs to decrease the intracellular levels of ROS and treat the injuries caused due to peroxidation. A study used Drosophila model and demonstrated that the dietary intake of Fe3O4 can increase the ability of climbing and longevity of Drosophila by decreasing the in vivo levels of ROS and reducing the risk of neurodegeneration and thus leads to the prevention of the Alzheimer's disease [209]. Moreover, the complexes of multienzymes can exhibit numerous types of therapeutic effects; for example, the latest nanocomposite which is composed of MnO2 NP (catalase and superoxide dismutase) and V2O5 nanowire (for glutathione peroxidase) demonstrates antioxidative properties for the significant elimination of both in vitro and in vivo ROS by employing an inflammation model of rodent [210]. Nowadays, the latest artificial hybrid metalloenzyme was synthesized for the treatment of hyperuricemia by enclosing platinum NPs (PtNPs) and uricase into the mesoporous silica NPs [211]. This model can demonstrate catalytic properties in which the uric acid and oxygen are converted to allantoin and H2O2 by uricase and the PtNPs catalyze the transformation of H2O2 to O2 and H20, lowering the toxic levels and supplying a uricase reagent. The in vivo studies have demonstrated effective therapeutic functions in mice without causing much toxicity during the treatment of hyperuricemia.  Figure 3 shows the nanomaterials applications in pharmacology, and Table 3 represents the list of approved NP-based therapeutic agents and imaging applications.

### 1.6. Conclusion and Future Prospects

NMs have demonstrated effective pharmacological, clinical, and biomedical applications and have become an interesting field for research and development. With its wide uses in drug design, drug development, imaging, diagnosis, therapy, and prevention of numerous diseases, the evaluation of toxicity of NMs is the most essential and demanding field for research. Naturally occurring NMs possess certain properties that can be less harmful to living beings. But the presence of toxic effects has been observed in the nanosized materials in living systems. Thus, both natural and engineered NMs are known to cause acute toxicity, and in addition to that, viral NPs and nanozymes have been extensively examined for their cytotoxic effects in order to define their applications and dosing regimen. Therefore, various laws and regulations have been enforced to eliminate/reduce the hazards or risks caused due to NMs in consumer products. There is a wide horizon for the development of novel therapeutic agents by utilizing NMs, as have been earlier approved by FDA. But there are various barriers that have to be eliminated so that the therapeutic effects and least toxicity can be achieved, like in the case of NPs that demonstrated unique enzymatic characteristics and kinetics on the basis of their microenvironment, and thus, it becomes difficult to control them. There needs to be a careful design of agents that are accessible to biological targets and devoid of drug resistance. However, rapid development in bioengineering and materials science has caused the formation of substances with improvised catalytic characteristics and specificity. Moreover, there are many substances that cause catalysis via external stimuli such as temperature and pH, to carry out their therapeutic mechanisms, ultimately eliminating the toxic compounds or releasing controlled in situ bioactive agents. These mechanisms, which require activation by external stimuli, cannot function in the usual physiological environment, and thus, they provide scope for the development of precise and targeted drugs. But, there needs to be a further investigation for utilizing clinically appropriate animal models and conducting clinical trials for extensive evaluation. The futuristic approach should work on accomplishing maximum effectiveness and least toxic effects for developing economical formulations in order to achieve long-term therapeutic benefits, which will be essential for clinical translation. The distinct features of NMs and NPs can be widely utilized for laying the platform for cost-effective, safe, sustainable, and efficacious therapeutic options for mankind.

## Figures and Tables

**Table 1:** Advantages and disadvantages of the methods of synthesis of NPs [63].

Methods	Advantages	Disadvantages
Top-down synthesis method	(i) Controlled distribution of particle size and morphology	(i) Costly method (ii) Complex technique
Bottom-up method	(i) Economical (ii) Controlled distribution of particle size and morphology	(i) Harmful technique as it consists of toxic residuals and ligands. (ii) Not environment friendly

**Table 2:** Preclinical studies of nano-based drug delivery platform for various diseases.

Indication	Animal model	NM-based drug delivery platform	Active pharmaceutical Ingredient	Outcomes	References
Hypertension	Male rats	PLA NPs	Aliskiren	Improved efficacy in terms of lowering blood pressure (BP) in NP-loaded aliskiren.	Pechanova et al. [99]
Hypertension	Wistar rats	Solid-lipid NPs	Isradipine	Isradipine-loaded NPs demonstrated a significant reduction in systolic BP till 36 hours.	Thirupathi et al. [100]
Atherosclerosis	Rabbit	Lipid NPs	Docetaxel	Significant decrease in atheroma area and expression of proinflammatory markers.	Meneghini et al. [101]
Atherosclerosis	ApoE null C57Bl/6 mice	Oligonucleotide functionalized NPs	-	Effective distribution to the atherosclerotic sit of the target	Sharma et al. [102]
Myocardial infarction	Male C57BL/6 mice	PLGA nanoparticles	Pitavastatin	Decrease in postinfarct left ventricular remodelling and monocytes and macrophages.	Mao et al. [103]
Myocardial infarction	Wistar rats	Lipid core NPs	Methotrexate	Significant reduction in infarction size, necrosis, expression of proinflammatory markers and improved systolic activities of left ventricle.	Maranhao et al. [104]
Myocardial ischemia	Bama minipigs	PLGA nanoparticles	Pitavastatin	Decrease in infarct size without causing any adverse events.	Ichimura et al. [105]
Myocardial ischemia	Male C57BL/6 mice	PLGA nanoparticles	Cyclosporine A	Improved cardioprotective actions and inhibition of mitochondrial permeability transition pore opening.	Ikeda et al. [106]

**Table 3:** List of approved NP-based therapeutic agents and imaging applications.

Product	Year of approval	Agency	Active pharmaceutical ingredient	Type of NP	Indication	References
Marqibo	2012	FDA	Vincristine	Lipid based	Acute lymphoid leukemia	Johnston et al. [212]; Krishna et al. [213]
Onivyde	2015	FDA	Irinotecan	Lipid based	Pancreatic Cancer, Colorectal Cancer	Drummond et al. [214]
Vyxeos	2018	EMA	Daunorubicin Cytarabine	Lipid based	Acute myeloid leukemia	Krauss et al. [215]
Pazenir	2019	EMA	Paclitaxel	NA	Metastatic breast cancer, metastatic adenocarcinoma of the pancreas, nonsmall cell lung cancer	Rodríguez et al. [216]
Rapamune	2015	FDA	Sirolimus (rapamycin)	Nanocrystals	a rare progressive lung disease (lymphangioleiomyomatosis)	Narayan et al. [217]
Abelcet	1995	FDA	Liposomal amphotericin B lipid complex	Lipid based	Fungal infections	Lister et al. [218]
AmBisome	1997	FDA	Liposomal amphotericin B	Lipid based	Fungal/protozoal infections	Lister et al. [218]
Onpattro	2018	FDA EMA	Patisiran	Lipid based	hereditary transthyretin (TTR) mediated amyloidosis	Weng et al. [219]; Huang et al. [220]
Pfizer-BioNTech vaccine	2020	FDA	mRNA vaccine	Lipid based	prevents COVID-19 infection	Oliver et al. [221]; Meo et al. [222]
Moderna COVID-19 vaccine	2020	FDA	mRNA vaccine	Lipid based	prevents COVID-19 infection	Meo et al. [222]; Hinton et al. [223]
Oncaspar	2016	EMA	L-asparaginase	Polymer based	acute lymphoblastic leukemia, chronic myelogenous leukemia	Harris and Chess. [224]
Mircera	2018	FDA	Epoetin *β*	Polymer based	Anemia	McGahan. [225]
Plegridy	2014	FDA	recombinant IFN-*β*	Polymer based	Relapsing-remitting multiple sclerosis (RRMS) in adult patients	Chaplin et al. [226]
Adynovate	2015	FDA	Coagulation factor VIII	Polymer based	Hemophilia A	Turecek et al. [227]
Rebinyn	2017	FDA	Recombinant DNA-derived coagulation FIX	Polymer based	Hemophilia B	Ezban et al. [228]; Nielsen et al. [229]
Zilretta	2017	FDA	Triamcinolone acetonide	Polymer based	knee osteoarthritis	Rai et al. [230]
VivaGel BV	2015	FDA	Astodrimer sodium	Dendrimer based	anti-infective for prevention of recurrent bacterial vaginosis	Madan et al. [231]
Hensify	2019	EMA	Hafnium oxide nanoparticles	Inorganic	locally advanced squamous cell carcinoma	Germain et al. [232]
Feridex (discontinued)	1996	FDA	SPION-dex	Inorganic	Imaging agent	Wang et al. [233]
Umi (discontinued)	2009	FDA	SPION-silicone	Inorganic	Imaging agent	Wang et al. [233]; Gil et al. [234]

FDA: Food and Drug Administration; EMA- European Medicines Agency.

## Data Availability

All data used to support the findings of this study are included in the article.
